# Insecticide resistance and role in malaria transmission of *Anopheles funestus* populations from Zambia and Zimbabwe

**DOI:** 10.1186/s13071-014-0464-z

**Published:** 2014-10-08

**Authors:** Kwang S Choi, Riann Christian, Luisa Nardini, Oliver R Wood, Eunice Agubuzo, Mbanga Muleba, Shungu Munyati, Aramu Makuwaza, Lizette L Koekemoer, Basil D Brooke, Richard H Hunt, Maureen Coetzee

**Affiliations:** Wits Research Institute for Malaria, School of Pathology, Faculty of Health Sciences, University of the Witwatersrand, Johannesburg, South Africa; Centre for Opportunistic, Tropical and Hospital Infections, National Institute for Communicable Diseases, National Health Laboratory Service, Johannesburg, South Africa; School of Life Sciences, College of Natural Sciences, Kyungpook National University, Daegu, 702-701 Korea; Tropical Diseases Research Centre, Ndola, Zambia; Biomedical Research and Training Institute, Harare, Zimbabwe; National Institute for Health Research, Harare, Zimbabwe

**Keywords:** *Anopheles funestus*, mtDNA clades, Insecticide resistance, Resistance intensity, *Plasmodium* infection

## Abstract

**Background:**

Two mitochondrial DNA clades have been described in *Anopheles funestus* populations from southern Africa. Clade I is common across the continent while clade II is known only from Mozambique and Madagascar. The specific biological status of these clades is at present unknown. We investigated the possible role that each clade might play in the transmission of *Plasmodium falciparum* and the insecticide resistance status of *An. funestus* from Zimbabwe and Zambia.

**Methods:**

Mosquitoes were collected inside houses from Nchelenge District, Zambia and Honde Valley, Zimbabwe in 2013 and 2014. WHO susceptibility tests, synergist assays and resistance intensity tests were conducted on wild females and progeny of wild females. ELISA was used to detect *Plasmodium falciparum* circumsporozoite protein. Specimens were identified to species and mtDNA clades using standard molecular methods.

**Results:**

The Zimbabwean samples were all clade I while the Zambian population comprised 80% clade I and 20% clade II in both years of collection. ELISA tests gave an overall infection rate of 2.3% and 2.1% in 2013, and 3.5% and 9.2% in 2014 for Zimbabwe and Zambia respectively. No significant difference was observed between the clades. All populations were resistant to pyrethroids and carbamates but susceptible to organochlorines and organophosphates. Synergist assays indicated that pyrethroid resistance is mediated by cytochrome P450 mono-oxygenases. Resistance intensity tests showed high survival rates after 8-hrs continuous exposure to pyrethroids but exposure to bendiocarb gave the same results as the susceptible control.

**Conclusions:**

This is the first record of *An. funestus* mtDNA clade II occurring in Zambia. No evidence was found to suggest that the clades are markers of biologically separate populations. The ability of *An. funestus* to withstand prolonged exposure to pyrethroids has serious implications for the use of these insecticides, either through LLINs or IRS, in southern Africa in general and resistance management strategies should be urgently implemented.

## Background

In the recent past, malaria vector control has been primarily pyrethroid-based through indoor residual house spraying [IRS] or the use of long-lasting insecticide-treated bed nets (LLINs) or both. This intensive use of insecticides, together with pesticide usage in agriculture, has led to a dramatic increase in resistance in the mosquito vectors across the whole African continent [http://www.irmapper.com]. *Anopheles funestus*, one of the four major vectors of *Plasmodium falciparum* malaria in Africa [1,2], is widely distributed throughout much of the African tropics and subtropics [3]. The earliest records of insecticide resistance in *An. funestus* are from the 1980s [4] and currently resistance in this species is known from Mali, Guinea, Ghana, Benin, Uganda, Kenya, Malawi, Zambia, Mozambique and South Africa [1]. A well-documented impact of insecticide resistance on vector control programme failure is the major malaria epidemic that occurred in South Africa in 1999/2000 when pyrethroid resistant *An. funestus* returned to South Africa after DDT was replaced with pyrethroids for indoor house spraying [5]. The situation returned to pre-failure levels after DDT was reintroduced and used in a mosaic system together with pyrethroids. A number of insecticide resistance studies on *An. funestus* from southern Africa have been documented, but studies from other regions are limited [1].

*Anopheles funestus* belongs to a group of at least 11 species, all of which are morphologically similar at the adult stage [6,7]. Some members of the group can be distinguished on egg and larval characteristics (*An. confusus, An. rivulorum* and *An. leesoni*) but this is not always the case and those species belonging to the *An. funestus* sub-group (*An. funestus, An. funestus-*like, *An. parensis, An. aruni* and *An. vaneedeni*) are virtually identical [1,6,7]. The importance of species identification lies in the fact that of the 11 species, only *An. funestus* plays a major role in malaria transmission, this species being highly adapted to humans and human habitation with parasite infection rates sometimes as high as 22% [6]. The only other member of the group that has been implicated in low-level, localised transmission is *An. rivulorum* in Tanzania [8] and western Kenya [9] but in each case the parasite infection rates have been below 1%.

Malaria vector control programmes need to know that their interventions are targeting the major vectors so that scarce resources are not wasted on non-vector mosquitoes. As a result, various genetical techniques have been applied to members of the *An. funestus* group with the aim of providing accurate species identification. Initially, the banding patterns of the giant polytene chromosomes were used [10,11] but these have limited practical value because *An. funestus* and *An. vaneedeni* share the same inversion arrangements resulting in the same banding patterns [10], as do *An. funestus* and *An. funestus*-like [12]. In addition, the technique has the disadvantage of being applicable only to half-gravid female mosquitoes. However, studies of the chromosomal inversion polymorphisms in Burkina Faso revealed the existence of two distinct forms that apparently do not mate in sympatry [13] and that are also significantly differentiated at the molecular level [14], supporting the hypothesis of two distinct species being present. Twenty years after the work of Green and Hunt [10,11], a molecular assay was developed that allowed for the rapid and accurate identification of the five most common members of the *An. funestus* group [15] and this is the standard protocol used in many laboratories today.

While *An. funestus* has abundant levels of molecular and chromosomal polymorphism across its range, there are only a few population genetic studies of the species and these are limited in geographic scope compared with similar work on the *Anopheles gambiae* complex [1]. Phylogenetic studies using mitochondrial DNA (mtDNA) have revealed that there are two clades within *An. funestus*. Clade I is widespread throughout the continent and clade II is known only from southern Mozambique and Madagascar [16,17]. It is estimated that these two clades evolved independently about 1 million years ago based on fixed differences and divergence in mtDNA [16]. However, the deep mtDNA divergence was not accompanied by corresponding nuclear divergence as measured by 10 microsatellite loci in the same study [16] so it is not clear what this genetic variation means in terms of species differentiation.

In the present study, we investigated the biological attributes of insecticide resistance and parasite infection rates that both impact on malaria vector control activities, in relation to the mtDNA clades in Zambia and Zimbabwe.

## Methods

### Field collections

*Anopheles funestus* specimens were collected resting inside houses from Honde Valley, Zimbabwe [18° 23.161′S, 32° 59.946′E] and Nchelenge District, Zambia [9° 19.115′S, 28° 45.070′E]. Permission to enter houses was obtained from the village headmen and from each individual house owner. Collections were carried out between February and April 2013. In February and March 2014, the most productive houses identified from the 2013 survey, were visited with the intention of maximizing the sample size over the few days that were available for collecting.

### Insecticide susceptibility tests

The WHO insecticide resistance tests [18] were performed in the field (Zambia both years; Zimbabwe 2013 only, 2014 tests were carried out on F-1 progeny) to investigate the susceptibility status of wild-caught *An. funestus* females to WHO diagnostic doses of pyrethroids (0.05% deltamethrin and 0.05% lambda-cyhalothrin), carbamates (0.1% bendiocarb and 0.1% propoxur), organochlorines (4.0% DDT and 4.0% dieldrin) and organophosphates (5.0% malathion, 1.0% fenitrothion and 0.25% pirimiphos-methyl). The age of the mosquitoes used for the tests was unknown and the physiological status ranged from blood fed to gravid. Each exposure consisted of ~25 randomly selected mosquitoes for the seven insecticides plus controls. Exposure time for six of the insecticides was 1 hour while mosquitoes were exposed to fenitrothion for 2 hours [18]. The total number of mosquitoes exposed to each insecticide was dependent on the numbers of mosquitoes available in the field and the initial results obtained for each insecticide. Final mortality was recorded 24 hours post-exposure. Efficacy of WHO insecticide papers was confirmed before and after the field tests using a known susceptible mosquito strain (FANG - *An. funestus* originating from Angola and colonised in 2003). All exposed specimens were stored on silica gel and returned to the laboratory for further processing.

**Figure 1:**
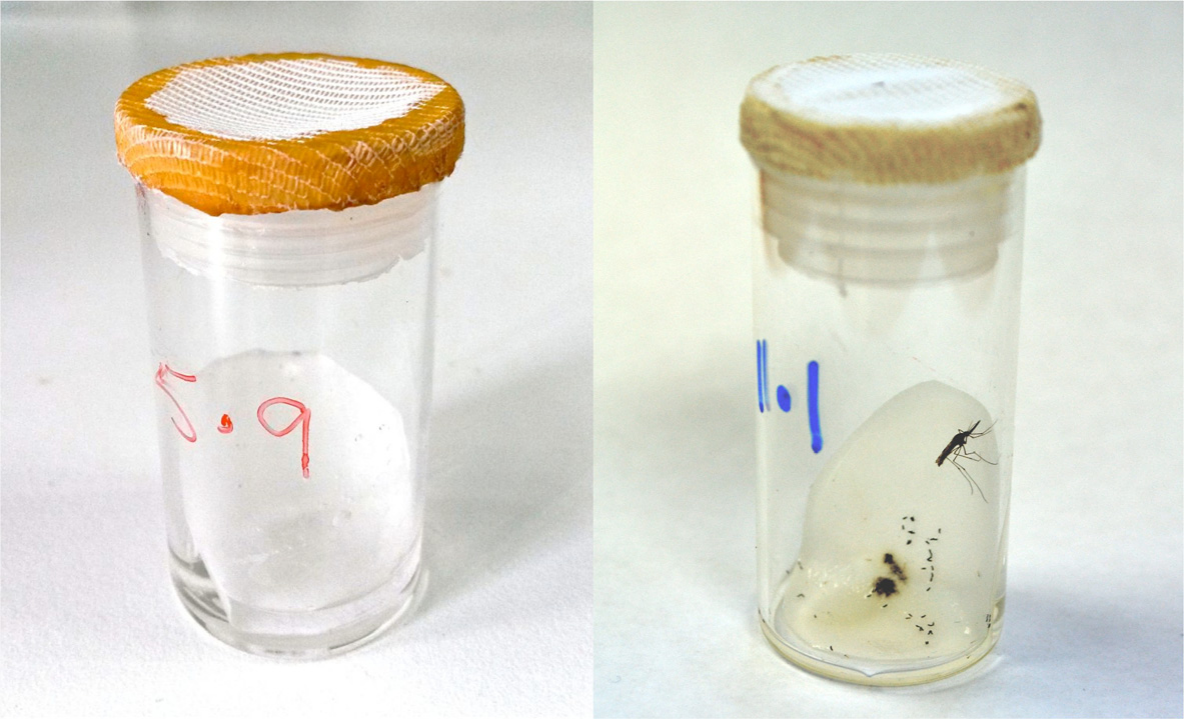
**A very simple method for inducing egg-laying in wild** ***Anopheles funestus*** **that consistently results in >70**% **of the females ovipositing.** Glass vials, 45 mm high x 25 mm diameter, with gauze lids are used. Small pieces of filter paper are placed at an angle in the bottom of the tube with approximately 1 ml water for egg laying. Females, resting inside the plastic lids, can be transferred to clean vials once they have laid eggs, facilitating blood-feeding for multiple egg batches.

A proportion of the collection from both localities was brought back to the laboratory alive. In the insectary, the wild females were set up for egg-laying in small glass vials lined with oval pieces of filter paper with a small amount of water in the bottom [9,19] (Figure 1) and held in wooden racks. This method usually results in >70% of wild females laying eggs. The Zimbabwe collections yielded 92 egg batches in 2013 and 140 in 2014. The Zambia collections yielded 148 clade I and 40 clade II egg batches in 2013, and 265 clade I and 67 clade II in 2014. Egg batches were reared individually until species/clade identification was obtained, at which point they were pooled into their separate groups. First generation adults, aged 2–5 days, were used for synergist and resistance intensity assays and, in the case of the 2014 Zimbabwe collections, also for WHO susceptibility tests.

### Resistance intensity assays

Female Zimbabwe and Zambia samples were exposed to 0.05% deltamethrin, 0.1% bendiocarb and 0.05% lambda-cyhalothrin (Zimbabwe samples only) treated papers continuously for 8 hours with knockdown being recorded at various time intervals. The WHO test tubes were laid flat so that once knocked down, the mosquitoes continued to be exposed to the insecticide. The 8-hr cut-off was arbitrarily chosen as the likely time a mosquito might come into contact with a sprayed wall after taking a blood meal. The susceptible laboratory strain FANG was used as a control.

### Synergist bioassays

Synergist assays [20] were performed by exposing 153 clade I specimens from Zimbabwe, 128 clade I and 113 clade II specimens from Zambia to 4% piperonyl butoxide (PBO), an inhibitor of monooxygenases, for 1 h prior to exposing them to deltamethrin and bendiocarb. Controls consisted of specimens exposed to the insecticides alone, and to PBO alone. The samples for the synergist assays were 2–3 day old unfed, F_1_ females.

### Laboratory analyses

Specimens were initially identified morphologically in the field using the keys of Gillies and Coetzee [7]. DNA was extracted from either legs or wings of individual mosquitoes using a *prep*GEM insect DNA extraction kit (ZyGEM, New Zealand) following the manufacturer’s instructions. Briefly, legs or wings of mosquitoes were homogenized with reagents, incubated for 15 minutes at 75°C and then for 5 minutes at 95°C. A total of 10 μL of solution from each sample was produced from the extraction procedure. All specimens were identified to species using the method of Koekemoer *et al*. [15]. After PCR species identification, a subsample of *An. funestus* was identified to clades using the hydrolysis probe analysis (Taqman assay) [21].

*Plasmodium falciparum* parasite infection in mosquitoes was detected from heads and thoraces of individual females using the enzyme-linked immunosorbent assay (ELISA) [22]. To validate the results, all of the positive samples were re-analyzed with the ELISA after heating the ELISA-homogenates in a heat block for 10 minutes at 100°C [23]. A positive control (recombinant *P. falciparum*) and seven negative controls (uninfected insectary female *An. funestus*) were included. An Ascent Multiskan (RC vl. 5.0, Genesis version 3.03, Labsystems) plate-reader was used for the analysis of data. The number of specimens that remained positive after heating the lysate was used to calculate the sporozoite rate.

### Data analysis

Resistance intensity assay data were compared using one-way ANOVA (Statistix 8 -Talahasee, Fl, USA). Variations in mortality between synergised and unsynergised samples were assessed using Chi-square (Statistix 8 -Talahasee, Fl, USA).

## Results

### Identification of *An. funestus*

All specimens were identified morphologically as belonging to the *An. funestus* group [7]. Table 1 presents the results of molecular species confirmation and clade identifications by year [15,21] of a total of 482 and 845 specimens of *An. funestus* from Zimbabwe and Zambia respectively. The Taqman assay used for mtDNA clade identification [21] revealed that only clade I was present in Zimbabwe, although sample sizes were relatively small and clade II might have been missed. In Zambia, 677 specimens were identified as clade I (80%) and 168 as clade II (20%). These proportions did not differ between 2013 and 2014 collections. A single specimen of *An. leesoni* was identified by PCR.

**Table 1:** **Identification of clades and** ***P. falciparum*** **infection rates by ELISA for** ***An. funestus*** **from Zimbabwe and Zambia**

	**n**	**Clades**		**ELISA**		
		**I**	**II**	**Clade I**	**Clade II**	**Total**
**Zimbabwe 2013**	342	92 (100%)	-	7/303 (2.3%)	-	7/303 (2.3%)
**Zimbabwe 2014**	140	88 (100%)	-	4/115 (3.5%)	-	4/115 (3.5%)
**Zambia 2013**	513	413 (80.5%)	100 (19.5%)	8/346 (2.3%)	1/76 (1.3%)	9/422 (2.1%)
**Zambia 2014**	332	264 (79.5%)	68 (20.5%)	17/171 (9.9%)	5/68 (7.4%)	22/239 (9.2%)

### Enzyme-linked immunosorbent assays (ELISA)

The *Plasmodium falciparum* infection rates are given in Table 1. A total of 725 female mosquitoes were tested using the original ELISA method [22] with a second re-analysis of positive specimens using the heated ELISA lysate method [23] to confirm the results. In 2013, the infection rates were 2.3% for clade I from both Zimbabwe and Zambia while the Zambian clade II samples had an infection rate of 1.3%. In 2014, both localities showed an increase in sporozoite rates with 3.5% in Zimbabwe, 9.9% in Zambia clade I and 7.4% in clade II.

### Insecticide resistance WHO bioassays

Insecticide resistance tests were carried out using the standard WHO methods and diagnostic doses [18] for two pyrethroids, two carbamates, two organochlorines and three organophosphates. *Anopheles funestus* from Zimbabwe and Zambia were all susceptible to the organochlorines and the organophosphates but resistant to pyrethroids and carbamates (Table 2). Table 3 gives the susceptibility results for the two clades in Zambia to pyrethroids and carbamates but given that the sample sizes are very small, these results require further investigation. In all cases, *An. funestus* meets the WHO criteria for resistance (i.e. <90% mortality) to both pyrethroids and carbamates [18].

**Table 2:** **Insecticide susceptibility tests of total** ***An. funestus*** **from Zimbabwe and Zambia for two consecutive years**

	**Zimbabwe**		**Zambia**	
	**%24 hr mortality (n) 2013**	**% 24 hr mortality (n) 2014**	**%24 hr mortality (n) 2013**	**% 24 hr mortality (n) 2014**
**Deltamethrin**	65.3 (49)	85.1 (94)	47.2 (72)	45.5 (99)
**Lambda-cyhalothrin**	24.1 (83)	3.7 (54)	19.0 (79)	-
**Bendiocarb**	70.1 (67)	73.1 (104)	75.5 (102)	45.2 (104)
**Propoxur**	77.4 (53)	-	60.5 (76)	79.3 (92)
**DDT**	100 (55)	100 (123)	100 (73)	100 (62)
**Dieldrin**	100 (43)	-	100 (68)	100 (56)
**Malathion**	100 (48)	-	100 (106)	99 (95)
**Fenitrothion**	100 (56)	-	100 (92)	100 (43)
**Pirimiphos-methyl**	-	100 (104)	-	100 (96)
**Controls**	0 (82)	1.9 (104)	0 (94)	4.8 (83)

**Table 3:** **Status of insecticide resistance for the clades of wild** ***An. funestus*** **females from Zambia in 2013**

	**Clade I**		**Clade II**	
	**Sample size**	**% 24 hr mortality**	**Sample size**	**% 24 hr mortality**
**Deltamethrin**	59	42.4	13	76.9
**Lambda-cyhalothrin**	60	18.3	19	21.0
**Bendiocarb**	83	77.5	19	73.7
**Propoxur**	67	57.6	9	77.3

### Resistance intensity experiments

**Figure 2:**
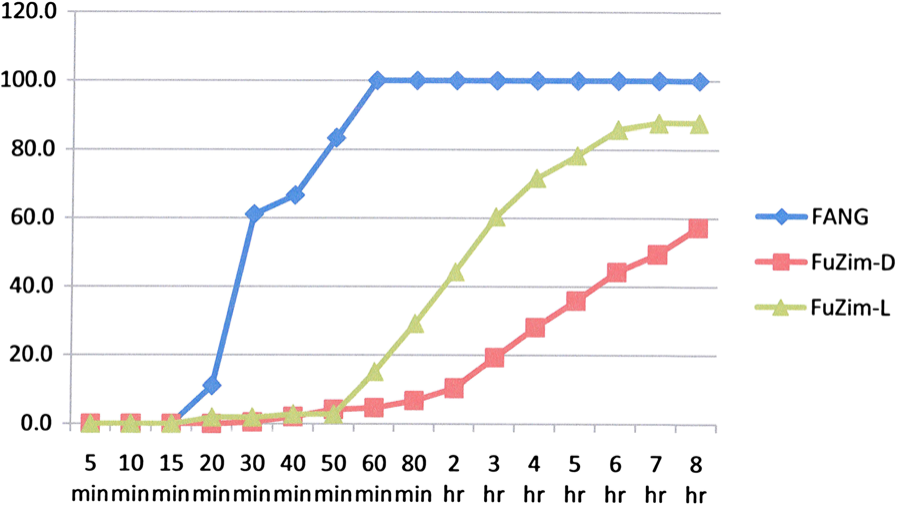
**Insecticide resistance intensity tests where susceptible (FANG) and Zimbabwe resistant** ***An. funestus*** **were exposed to 0.05%** **deltamethrin (FuZim-D) and lambda-cyhalothrin (FuZim-L) for 8 hours.**

**Figure 3:**
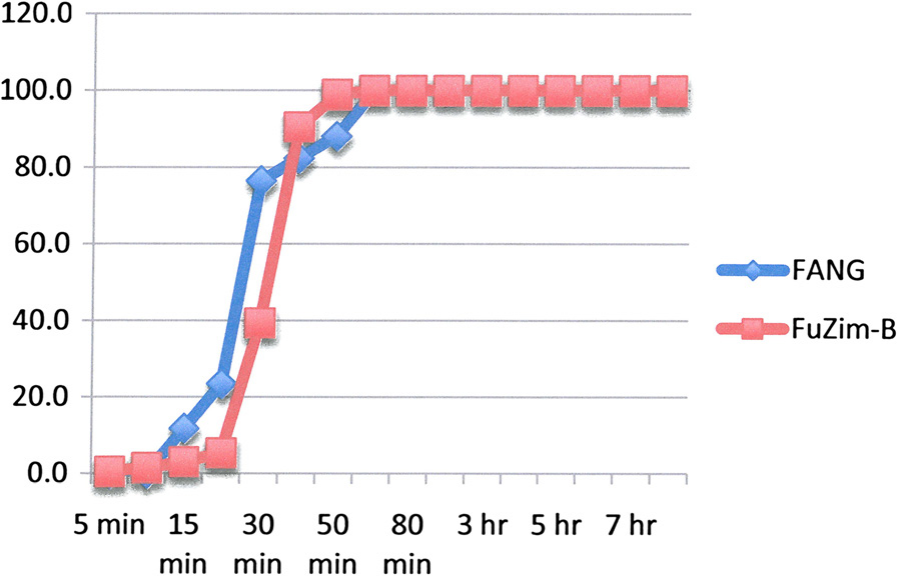
**Insecticide resistance intensity tests where susceptible (FANG) and Zimbabwe resistant (FuZim-B)** ***An. funestus*** **were exposed to 0.1% bendiocarb for 8 hours.**

**Figure 4:**
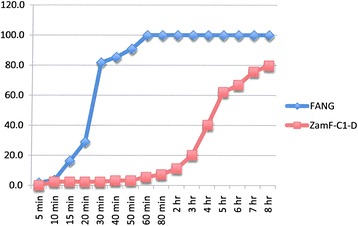
**Insecticide resistance intensity tests where susceptible (FANG) and Zambia resistant clade I (ZamF-C1-D)** ***An. funestus*** **were exposed to 0.05% deltamethrin for 8 hours.**

**Figure 5:**
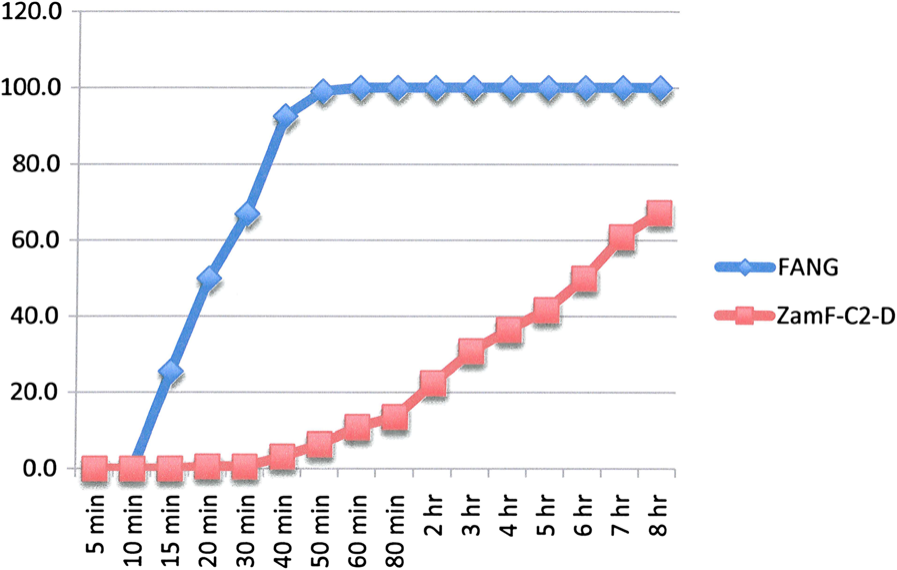
**Insecticide resistance intensity tests where susceptible (FANG) and Zambia resistant clade II (ZamF-C2-D)** ***An. funestus*** **were exposed to 0.05% deltamethrin for 8 hours.**

**Figure 6:**
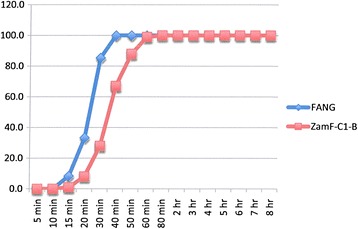
**Insecticide resistance intensity tests where susceptible (FANG) and Zambia resistant clade I (ZamF-C1-B)** ***An. funestus*** **were exposed to 0.1% bendiocarb for 8 hours.**

**Figure 7:**
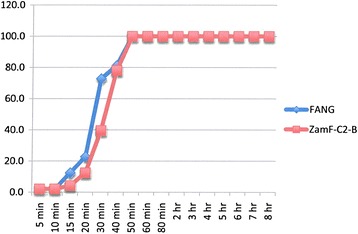
**Insecticide resistance intensity tests where susceptible (FANG) and Zambia resistant clade II (ZamF-C2-B)** ***An. funestus*** **were exposed to 0.1% bendiocarb for 8 hours.**

The results are shown in Figures 2, 3, 4, 5, 6, 7 and Table 4. Data points in Figures 2, 3, 4, 5, 6 and 7 are overall percentage knock-downs across all replicates. Both Zimbabwe clade I and Zambian clades I and II showed similar results for 8-hr exposures to deltamethrin that were significantly different to the FANG results (ANOVA: df = 1; P < 0.01 in all cases) (Figures 2, 4 and 5). There were sufficient numbers of Zimbabwe F_1_s to carry out tests on lambda-cyhalothrin and the results showed that there was no statistically significant difference between the responses to this insecticide compared with deltamethrin over the entire 8 hr monitoring period (ANOVA: df = 1; F = 3.33; P = 0.08), although the rate of knockdown induced by lambda-cyhalothrin was significantly higher than that induced by deltamethrin for the period 60 min to 8 hrs (ANOVA: df = 1; F = 6.55; P = 0.02) (Figure 2).

**Table 4:** **Resistance intensity assays of** ***Anopheles funestus*** **females by insecticide and strain/clade**

**Insecticide**	**Strain/clade**	**N (replicates)**	**Mean % knock-down (SE) after 8 hrs exposure**
Deltamethrin	FANG	179 (7)	100
	FUZIM	192 (8)	56.9 (3.9)
	ZAMF C1	124 (5)	80.9 (6.4)
	ZAMF C2	156 (6)	67.2 (1.9)
Bendiocarb	FANG	113 (5)	100
	FUZIM	183 (9)	100
	ZAMF C1	100 (4)	100
	ZAMF C2	96 (4)	100
Lambda-cyhalothrin	FANG	18 (1)	100
	FUZIM	106 (4)	87.9 (3.1)

Mean percentage knock-downs after 8 hour exposures are shown with standard errors (SE) in parentheses where appropriate. FANG = insecticide susceptible laboratory strain; FUZIM = F1 progeny from wild-caught females from Zimbabwe; ZAMF C1 = F1 progeny from wild-caught clade I females from Zambia; ZAMF C2 = F1 progeny from wild-caught clade II females from Zambia; N = sample size with number of replicates in parentheses.

Experiments carried out on bendiocarb showed no difference between either locality compared with each other or with their corresponding susceptible FANG (ANOVA: df = 1; P > 0.05 in all cases) (Figures 3, 6 and 7).

### Synergist bioassays

These assays [20] were performed on 2–5 day old F_1_ progeny from both Zambia and Zimbabwe using piperonyl butoxide (PBO) as the synergist for P450 mono-oxygenase enzymes. Table 5 summarises the results of the synergist bioassays. Pre-exposure to PBO completely nullified deltamethrin resistance in all three samples/clades (Chi-square: df = 2; X^2^ = 7.83; P = 0.02), and almost completely nullified bendiocarb resistance in all three samples/clades (Chi-square: df = 2; X^2^ = 43.8; P < 0.01).

**Table 5:** **Synergist experiments on** ***An. funestus*** **clades I and II from Zimbabwe (Honde Valley) and Zambia (Nchelenge)**

	**Zimbabwe Clade I % mortality (n)**	**Zambia Clade I % mortality (n)**	**Zambia Clade II % mortality (n)**
**Deltamethrin**	44.0 (50)	19.2 (26)	29.6 (54)
**Deltamethrin + PBO**	100 (97)	100 (84)	100 (67)
**Bendiocarb**	85.2 (57)	6.7 (45)	34.0 (47)
**Bendiocarb + PBO**	100 (56)	84.1 (44)	84.8 (46)
**Control PBO**	5.1 (79)	3.8 (52)	-

% mortality = percentage mortality 24-hr post-exposure. (n) = number of mosquitoes exposed.

## Discussion and conclusions

Our data show that *An. funestus* clade II is not confined to southern Mozambique and Madagascar [16,17] and can be found as far north as Nchelenge District in Zambia, being 2,000 km further north than the current distribution of Chibuto in Mozambique [17]. We do not have data on seasonal abundance of the two clades, so it is possible that clade II also occurs in the Honde Valley, Zimbabwe, but was not detected due to its very low frequency at both times of collection. The frequency of clades I and II in Nchelenge District remained stable over the two years of sampling. Neither the insecticide resistance data nor the sporozoite infection rates provided clear evidence that the clades are anything other than a polymorphism in a single panmictic population.

The *Plasmodium falciparum* infection rates for the 2013 Zimbabwe (2.3%) and Zambia (2.1%) populations were similar overall, with no significant difference between clade I (8/346 - 2.3%) and clade II (1/76 - 1.3%) (Pearson’s chi-square = 0.34, P = 0.56). However, there was a 4-fold increase in sporozoite rates in Nchelenge District in 2014, corresponding with an increase in reported malaria cases (ICEMR data, personal communication). A slight increase was also seen in the 2014 Zimbabwe sample. Of the eight households sampled almost every single inhabitant had had at least one bout of malaria during the 2013–2014 summer. This recent increasing trend of infective (and resistant) *An. funestus* being associated with increased number of malaria cases has been reported in other countries, such as Tanzania [24], Kenya [25] and Senegal [26]. These studies underline the importance of *An. funestus* as a major malaria vector that is sometimes underestimated by researchers and control programmes in Africa.

At present, malaria vector control programmes in Africa rely heavily on chemical methods, with long-lasting insecticide treated bed nets (LLINs) and indoor house spraying with residual insecticides (IRS) being the most widely implemented. However, the alarming increase in insecticide resistance in the main malaria vectors to many of the insecticides used in control, such as pyrethroids, carbamates and DDT, poses a very serious concern [27,28]. The susceptibility tests clearly showed that both these *An. funestus* populations are highly resistant to pyrethroids and carbamates while remaining susceptible to DDT and the organophosphates. The malaria vector control interventions at both localities include distribution of LLINs and in the Honde Valley, the houses had been sprayed with lambda-cyhalothrin in December 2013. Neither intervention had any apparent impact on malaria transmission.

The so-called resistance intensity test carried out here was not a standard method of measuring resistance. While the CDC bottle bioassay method [29] recommends that exposures be continued beyond the diagnostic time in order to assess resistance intensity, they recommend that experiments be terminated at 2 hours and the manual [29] does not give criteria for evaluating resistance intensity. At the 2-hr interval, both *An. funestus* populations showed mortality of less than 20% on the WHO diagnostic dose of 0.05% deltamethrin indicating an extremely high level of resistance. What this means in the field, however, is difficult to judge since these tests do not take into account repellent effects or whether an individual mosquito will indeed rest continuously for 8 hours, or even 1 hour on a treated surface. What is clear however, is that pyrethroid-resistant *An. funestus* is quite capable of withstanding prolonged exposure to the pyrethroids used for treating bed nets and for house spraying.

The problem of insecticide resistance in *An. funestus* is no longer a localized one. The resistant population first detected in South Africa [30] and southern Mozambique [20] has now spread to northern Malawi [31] and is affecting the region as a whole, including the localities sampled in this study. The evidence that we have indicates that the resistance mechanisms are mediated by metabolic monooxygenase enzymes, well documented in other *An. funestus* populations [1]. This metabolic system does not incur a fitness cost [32] and even in the absence of insecticide pressure, the resistance is not lost, facilitating the geographical spread of resistance alleles. However, the resistance in *An. funestus* populations is by no means uniform across southern Africa with recent studies in south-eastern Zambia showing resistance to DDT as well as pyrethroids and carbamates [33].

This places yet more constraints on the vector control programmes operating in the region with only the organophosphates having a generalised killing effect on the *An. funestus* populations. While both Zambia and Zimbabwe are changing their IRS policies and will be using an organophosphate for IRS in the coming transmission season, the need for additional, affordable, new tools for vector control [28] has acquired an urgency as never before.
